# Parasite responses to pollution: what we know and where we go in ‘Environmental Parasitology’

**DOI:** 10.1186/s13071-017-2001-3

**Published:** 2017-02-06

**Authors:** Bernd Sures, Milen Nachev, Christian Selbach, David J. Marcogliese

**Affiliations:** 1 Aquatic Ecology and Centre for Water and Environmental Research, University of Duisburg-Essen, Universitätsstr. 5, D-45141 Essen, Germany; 2 Department of Zoology, University of Johannesburg, PO Box 524, Auckland Park 2006, Johannesburg, South Africa; 3 Water Research Group, Unit for Environmental Sciences and Management, North-West University, Private Bag X6001, Potchefstroom, 2520 South Africa; 4 Aquatic Contaminants Research Division, Water Science and Technology Directorate, Science and Technology Branch, Environment and Climate Change Canada, St. Lawrence Centre, 105 McGill Street, 7th floor, Montreal, QC H2Y 2E7 Canada; 5 St. Andrews Biological Station, Fisheries and Oceans Canada, 531 Brandy Cove Road, St, Andrews, NB E5B 2 L9 Canada

**Keywords:** Metal pollution, Ecosystem health, Biomarker, Ecotoxicology, Parasites, Endocrine disruption, Bioindication

## Abstract

Environmental parasitology deals with the interactions between parasites and pollutants in the environment. Their sensitivity to pollutants and environmental disturbances makes many parasite taxa useful indicators of environmental health and anthropogenic impact. Over the last 20 years, three main research directions have been shown to be highly promising and relevant, namely parasites as accumulation indicators for selected pollutants, parasites as effect indicators, and the role of parasites interacting with established bioindicators. The current paper focuses on the potential use of parasites as indicators of environmental pollution and the interactions with their hosts. By reviewing some of the most recent findings in the field of environmental parasitology, we summarize the current state of the art and try to identify promising ideas for future research directions. In detail, we address the suitability of parasites as accumulation indicators and their possible application to demonstrate biological availability of pollutants; the role of parasites as pollutant sinks; the interaction between parasites and biomarkers focusing on combined effects of parasitism and pollution on the health of their hosts; and the use of parasites as indicators of contaminants and ecosystem health. Therefore, this review highlights the application of parasites as indicators at different biological scales, from the organismal to the ecosystem.

## Background

In recent years, research on environmental implications of parasites has seen a strong increase, leading to the establishment of ‘Environmental Parasitology’ (EP) as an accepted discipline covered in parasitology textbooks [1]. EP in the sense of an ecologically based approach focuses on parasites as indicators of environmental health. Occasionally, EP is also used in a medical context, especially when the contamination and occurrence of infective parasitic stages in the environment is addressed [2]. However, the current paper focuses on the function parasites may have as indicators of environmental quality. Following a number of influential reviews [3–15], many case studies were initiated to unravel possible impacts of anthropogenic changes on parasites. Among the variety of studies, the following three main research directions have been proven to be most promising: (i) parasites as accumulation indicators for selected pollutants, (ii) parasites as effect indicators in the broadest sense, and (iii) parasites interfering with the health of their hosts and with established monitoring or effect studies using free-living organisms. As these research directions have frequently been reviewed in the past (e.g. [12, 13, 16–18]) we intend to summarize the most recent findings in the field of pollution associated EP and try to identify promising ideas for future research.

The use of parasites as accumulation indicators specifically addresses the questions if and how parasites can be used to indicate the biological availability of certain substances which are commonly accepted to be harmful to the environment. Based on the fact that certain groups of endoparasites are excellent accumulators of toxic metals [12, 16, 19] and selected organic pollutants [20], one can suggest adding parasites to the list of already existing (free-living) accumulation indicators. As free-living species are usually much easier to work with than parasites, which are hidden in their hosts, good arguments are required to justify parasites as additional accumulation indicators. One such argument can be the proof of the biological availability of pollutants in those groups of parasites which lack a digestive system. If, for example, substances can be detected in acanthocephalans and cestodes, they had to cross through the parasites’ tegument and membranes and therefore have to be biologically available. In contrast, if substances are detected in filter-feeding organisms, such as mussels, it remains unclear if the substances are only loosely attached to the gills or present in the content of the intestine instead of being taken up on a cellular level. Additionally, the accumulation of toxic substances in parasites may also have implications for pollutant levels in the host tissues. We therefore have reviewed recent studies on possible beneficial effects [21] parasites may have on their hosts.

By definition, parasites are not neutral with respect to their interaction with their hosts. They have long been recognized as important pathogens of man and livestock which resulted in a growing body of knowledge on adverse effects parasites have on their hosts. Many of these are documented in medical and veterinary text books. In recent years however a variety of molecular tools has allowed us to get a more detailed understanding of the physiological and molecular interaction of parasites with their hosts. These interactions affect the physiological homeostasis of the host, often leading to negative effects on its health. Moreover, deviations from physiological homeostasis also occur if organisms are confronted with pollutants. In the field of ecotoxicology many of these deviations are used as biological markers to indicate effects of pollutants [17, 18]. The studies reviewed here show that unpredictable or contradictory results emerge if infected animals are used in ecotoxicological research without considering possible effects of parasites on biomarker responses.

Effect indication with parasites is a much more intricate field in EP, as it usually concentrates on complex biotic responses. In classical ecotoxicological research physiological, behavioral or molecular changes are determined as a response to adverse environmental changes, often due to the presence and effects of pollutants [22] or habitat disturbance. If parasites are considered as effect indicators, applicable approaches mainly focus on direct effects of pollutants on the viability and longevity of free-swimming stages such as cercariae or on changes in population and community structure. In the sense that parasites are integrative parts of food webs within ecosystems, environmental changes can be earmarked by parasites if one of their developmental stages or one of their hosts is negatively affected. In either situation, such adverse effects result in numerical changes of parasites, i.e. in changes of biodiversity patterns and associated indices, such as measures of diversity or the ratio between monoxenic and heteroxenic species. Once we are able to predict and calibrate such numerical changes within parasite communities depending on the type and intensity of human impacts, parasites can be powerful tools to indicate environmental changes. Recent studies on these issues are summarized and promising research ideas are presented and discussed.

In detail, we will address the following topics: (i) parasites as accumulation indicators and their possible application to demonstrate biological availability of pollutants; (ii) parasites as pollutant sinks; (iii) the interaction between parasites and biomarkers and their consequences for host health; (iv) contaminant effects on free-living stages of parasites; and (v) parasites as indicators for ecosystem health.

## Parasites as accumulation indicators and tools to demonstrate biological availability of pollutants

A large number of studies have demonstrated and highlighted a high accumulation potential of different parasite taxa and identified them as useful sentinels for chemical pollution. Table 1 provides a detailed summary of studies on metal accumulation in different parasite taxa. In comparison to established free-living accumulation indicators, parasites are often able to take up chemicals (e.g. metals) at much higher levels [12, 16–19]. Thus, they can bioconcentrate pollutants which are present in very low concentrations in the environment and make them detectable and quantifiable using conventional analytical techniques. Furthermore, some parasites were found to tolerate very high pollutant burdens (see below), which suggest that they might be applicable as sentinels for polluted habitats. Moreover, since accumulation indicators provide important information about the biological availability of pollutants, parasites represent possible diagnostic tools for assessing the behaviour of chemicals in the environment and to what degree they are available for uptake by the biota.

**Table 1:** Summary of the studies on metal accumulation in parasites published after the review paper of Sures [12]. Elements marked in bold were accumulated to a higher degree in the parasites than in the host tissues; ranges of bioconcentration factors with reference to host tissues were provided only for these elements

Habitat	Parasite taxa	Host	Host tissue	Element	Study type	BCF range	Reference
	Acanthocephala						
limnetic	*Acanthocephalus anguillae*	*Perca fluviatilis*	l	**Ag**, **Cd**, **Cu**, Fe, **Mn**, **Pb**	field	2.2–29.1	[157]
		*Squalius cephalus*	i	**Ag**, **Cd**, **Cu**,**Fe**, **Mn**, **Pb**, Zn	field	1–29.1	[158]
limnetic	*Acanthocephalus lucii*	*Perca fluviatilis*	m, l, go	**Pb**	field	9–55	[159]
		*Perca fluviatilis*	m, l, k, hr, br	**As**, Cd, **Cr**, **Cu**, Hg **Mn**, **Ni**, **Pb**, Zn	field	1.3–170.7	[160]
		*Perca fluviatilis*	m, go	Hg	field	BCF < 1	[161]
		*Perca fluviatilis*	m, l, k, hr, br	**As**, **Cd**, **Cr**, **Cu**, Hg **Mn**, Ni **Pb**, Zn	field	1.2–370	[162]
		*Perca fluviatilis*	m, l, go	**Cd**, **Cu**, **Mn**, **Zn**	field	2.2–194	[163]
limnetic	*Acanthogyrus* sp.	*Oreochromis niloticus*	m, i,l	**Pb**	field	102–147	[164]
terrestrial	*Moniliformis moniliformis*	*Rattus rattus*	l, k	**Cd**, **Pb**	field	1.2–86.9	[35]
		"urban rat"	m, l, k	**Cd**, **Cr**	field	4.7–17.1	[165]
limnetic	*Pomphorhynchus laevis*	*Barbus barbus*	m, i,l	**As**, **Cd**, **Co**, **Cu**, Fe, **Mn**, Mo, Ni, **Pb**, Sn, V, **Zn**	field	1.2–1,070	[166]
		*Barbus barbus*	m, i,l	**As**, **Cd**, **Co**, **Cu**, Fe, **Mn**, **Pb**, Se, Sn, V, **Zn**	field	1.2–337	[27]
		*Perca fluviatilis*	l	**Ag**, **Cd**, **Cu**, Fe, **Mn**, **Pb**	field	1.9–57.6	[157]
		*Squalius cephalus*	i	**Ag**, **Cd**, **Cu**,Fe, **Mn**, **Pb**, Zn	field	1.3–112.5	[158]
	Cestoda						
marine	*Anthobothrium* sp.	*Carcharhinus dussumieri*	m, i, l, go	**Cd**, **Pb**	field	21.4–1,175	[25]
limnetic	*Bathybothrium rectangulum*	*Barbus barbus*	m	Cd, **Cr**, **Ni**, **Pb**	field	1.2–2.3	[167]
limnetic	*Bothriocephalus acheilognathi*	*Labeobarbus kimberleyensis*	m, l, sc	As, Ba, **Be**, Cd, Co, Cr, Cu, Fe, **Hg**, **Li**, **Mn**, Mo, Ni, **Pb**, **Se**, Sb, Sn, Te, Ti, **Tl**, **U**, V, Zn,	field	na	[168]
limnetic	*Caryophyllaeus laticeps*	*Chondrostoma nasus*	m, i, l, gi	**Cd**, Cu, **Pb**, **Zn**	field	3–9.7	[169]
marine	*Clestobothrium crassiceps*	*Merluccius merluccius*	m, l, k	As, Hg, Se	field	BCF < 1	[170]
terrestrial	*Gallegoide sarfaai*	*Apodemus sylvaticus*	m, l, k	**Cd**, **Pb**	field	6.2–24	[171]
marine	*Gyrocotyle plana*	*Callorhinchus capensis*	m, i, l, k, go	Al, **As**, Cd, Co, Cr, Cu, **Mn**, Ni, **Pb**, Sb, Se, Sn, Th, **Ti**, U, V, **Zn**	field	1.1–23.4	[172]
terrestrial	*Hymenolepis diminuta*	"urban rat"	m, l, k	**Cd**, **Cr**	field	2.7–11.6	[165]
		*Meriones libycus*	i, l, k	**Pb**	field	7.55–21.9	[173]
		*Rattus norvegicus*	m, i, l, k, bo, te	**Pb**	experimental	2.6–210	[174]
marine	*Lacistorhynchus dollfusi*	*Citharichthys sordidus*	m, i, l	Ag, As, Cd, **Cr**, **Cu**, **Fe**, **Hg**, K, **Pb**, Rb, Se, **Sr**, **Ti**, **Zn**	field	1.9–117.6	[175]
limnetic	*Ligula intestinalis*	*Rastreneobola argentea*	whole fish	**Cd**, **Cr**, **Cu**, **Pb**	field	2.5–18	[46]
		*Tinca tinca*	m, l, go	Cd, Cr, **Cu**, **Fe**, **Mn, Zn**, Pb	field	1.6–37.4	[176]
		*Tinca tinca*	l	**Al**, B, **Ba**, Cd, Cr, Ni, Pb, **Sr**	field	1.2–3	[177]
		*Abramis brama*, *Blicca bjoerkna*, *Rutilus rutilus*	m	**Cd**, **Cr**, Ni, **Pb**	field	2.3–35.6	[167]
terrestrial	*Mesocestoides* spp.	*Vulpes vulpes*	l, k	**Cu**, **Cr**, **Mn**, **Ni**, **Pb**, **Zn**	field	1.9–52	[178]
terrestrial	*Moniezia expansa*	*Ovis aries*	m, l, k	**Pb**	experimental	4.0–458.5	[179]
terrestrial	*Moniezia expansa*	*Ovis aries*	m, k	**Cd**	experimental	1.5–31	[180]
terrestrial	*Mosgovoyia ctenoides*	*Oryctolagus cuniculus*	i, l, k	**As**, Cd, **Pb**, Hg	field	1.36–2.58	[181]
terrestrial	*Paranoplocephala dentata*	*Clethrionomys glareolus*, *Microtus agrestris*	l, k	Cd, Cr, Cu, **Mn**, **Ni**, **Pb**, **Zn**	field	1.7–37	[182]
marine	*Paraorigmatobothrium* sp.	*Carcharhinus dussumieri*	m, i, l, go	**Cd**, **Pb**	field	410–1,112.9	[25]
marine	*Polypocephalus* sp.	*Himantura* cf. *gerarrdi*	m, i	**Cd**, **Pb**	field	5.2–6.1	[183]
limnetic	*Proteocephalus macrocephalus*	*Anguilla anguilla*	m, l, k	As, Cd, **Cr**, Cu, Hg, **Ni**, **Pb**, Pd, Pt, **Zn**	field	2.1–15.8	[184]
limnetic	*Proteocephalus percae*	*Perca fluviatilis*	m, l, k, hr, br	**As**, Cd, **Cr**, Cu, Hg, **Mn**, Ni, **Pb**, **Zn**	field	1.8–149.0	[160]
		*Perca fluviatilis*	m, l, k, hr, br	**As**, Cd, **Cr**, Cu, Hg, **Mn**, **Ni**, **Pb**, Zn	field	1.7–234	[162]
terrestrial	*Raillietina micracantha*	*Columba livia*	m, l, k, fe	As, Cd, Cr, Cu, Hg, **Mn**, **Pb**, Se, Zn	field	6.1–79.8	[185]
marine	*Rhinebothrium* sp. 1	*Himantura* cf. *gerarrdi*	m, i	**Cd**, **Pb**	field	1.2–2.5	[183]
marine	*Rhinebothrium* sp. 2	*Glaucostegus granulatus*	m, i	**Cd**, **Pb**	field	2.4–3.7	[183]
terrestrial	*Rodentolepis microstoma*	*Mus domesticus*	m, l, k	**Cd**, **Pb**	field	1.2–60.6	[35]
limnetic	*Senga parva*	*Channa micropeltes*	m, i, l, k	Cd, Cu, **Mn**, **Pb**, **Zn**	field	na	[186]
terrestrial	*Skrjabinotaenia lobata*	*Apodemus sylvaticus*	m, l, k	Cd, **Pb**	field	8.5–81.4	[187]
terrestrial	*Taenia taenaeiformis*	"urban rat"	m, l, k	**Cd**, **Cr**	field	2.7–11.6	[165]
marine	*Tatragonocephalum* sp.	*Himantura* cf. *gerarrdi*	m, i	**Cd**, **Pb**	field	1.6–1.8	[183]
terrestrial	*Tetrabothrius bassani*	*Morus bassanus*	m, l, k	As, **Cd**, Cr, Cu, Hg, Mn, **Pb**, Se, Zn	field	6.9–9.5	[188]
	Nematoda						
limnetic	*Aguillicola crassus*	*Anguilla anguilla*	m, l,k	As, Cd, Cr, Cu, Hg, **Ni**, Pb, Pd, Pt, Zn	field	1.31	[184]
		*Anguilla anguilla*	m, l, sb, sk	Cd, Cr, Cu, **Fe**, Hg, Mn, Pb, Zn	field	25.5	[189]
marine	*Anisakis* sp.	*Dicentrarchus labrax*	m, l, gi	**Cd**, **Cu**, **Fe**, **Mn**, **Ni**, **Pb**, **Zn**	field	2–16	[190]
marine	*Ascaris* sp.	*Liza vaigiensis*	m, i	**As**, **Cd**, **Fe**, **Hg**, **Pb**, **Zn**	field	26.5–400	[191]
terrestrial	*Contracaecum* spp.	*Phalacrocorax auritus*	m	**Hg**	field	1.4	[192]
limnetic		*Acestrorhynchus lacustris*	m, l	**Al**, **As**, **Ba**, **Cd**, **Cu**, **Cr**, Fe, Mg, Mn, **Ni**, **Pb**, **Ti**, **Zn**	field	4.1–98.2	[193]
marine	*Dichelyne minutus*	*Chasar bathybius*	i, l	**Cu**, **Zn**	field	19–194	[28]
marine	*Echinocephalus* sp.	*Liza vaigiensis*	m, i	**As**, **Cd**, **Fe**, **Hg**, **Pb**, **Zn**	field	20.6–360	[191]
limnetic	*Eustrogylides* sp.	*Barbus barbus*	m, i, l	As, Cd, **Co**, **Cu**, **Fe**, Mn, **Pb**, **Se**, **Sn**, V, **Zn**	field	1.4–123	[27]
marine	*Hysterothylacium* sp.	*Trichiurus lepturus*	m, i, l, go	**Cd**, **Pb**	field	1.4–1,173.5	[194]
marine	*Hysterothylacium aduncum*	*Pagellus erythrinus*	m, i, l, sb, sk	Cd, **Cr**, Cu, Fe, **Hg**, Mg, Mn, **Pb**, Zn	field	1.1–113.9	[195]
		*Sparus aurata*	m, i, l, gi, sk	**Cd**, Cr, **Cu**, Fe, Hg, Mg, Mn, **Pb**, Zn	field	1.63–7.31	[196]
		*Solea solea*	m, l, gi, k	**Cd**, **Cu**, **Fe**, Ni, Pb, Zn	field	1.27–80	[197]
marine	*Hysterothylacium reliquens*	*Nemipterus peronii*	m, l, k	**Al**, **As**, **Cd**, **Cr**, **Cu**, **Fe**, Hg, **Mn**, **Ni**, **Pb**, **Se**, **Sr**, **Zn**	field	1.6–185	[198]
marine	*Paraphilometroides nemipteri*	*Nemipterus peronii*	m, l, k	**Al**, **As**, **Cd**, **Cr**, **Cu**, **Fe**, Hg, **Mn**, **Ni**, **Pb**, **Se**, **Sr**, **Zn**	field	1–1,861.2	[198]
limnetic	*Philometra ovata*	*Gobio gobio*	m	**Cd**, **Cr**, **Cu**, **Pb**, **Ni**, **Zn**	field	3.2–121.7	[199]
limnetic	*Procamallanus* spp.	*Synodontis clarias*	i	**Cd**, **Fe**, Mn, **Pb**, Zn	field	1.4–22.2	[200]
marine	*Proleptus obtusus*	*Rhinobatos annulatus, Rhinobatos blochii*	m, i, l, k, go	Al, As, Cd, Co, Cr, Cu, Mn, Ni, Pb, Sb, Se, Sn, Th, Ti, U, V, Zn	field	BCF < 1	[172]
terrestrial	*Toxascaris leonina*	*Vulpes vulpes*	l, k	Cu, **Cr**, **Mn**, **Ni**, **Pb**, **Zn**	field	1.2–7.7	[178]
terrestrial	*Brevimulticaecum tenuicolle, Dujardinascaris waltoni, Eustrongylides* sp., *Goezia* sp., *Ortleppascaris antipini*, *Terranova lanceolata*	*Alligator mississippiensis*	l	**As**, **Cd**, **Cu**, Fe, Pb, **Se**, **Zn**	field	1–102	[30]
	Digenea						
terrestrial	*Drepanocephalus spathans*	*Phalacrocorax auritus*	m	**Hg**	field	1.3	[192]
terrestrial	*Fasciola gigantica*	buffaloes	m, l	Cd, Cr, **Cu**, **Pb**, Zn	field	1.5–4.7	[31]
terrestrial	*Fasciola hepatica*	buffaloes	m, l	Cd, Cr, **Cu**, **Pb**, Zn	field	1.8–3.6	[31]
marine	*Neoapocreadium chabaudi*	*Balistes capriscus*	m, l, k	Se, Hg	field	BCF < 1	[201]
marine	*Robphildollfusium fractum*	*Sarpa salpa*	m, l, k	**Se**, Hg	field	1.2–7.15	[201]
limnetic	*Siphodera* spp.	*Chrysichthys nigrodigitatus*	i	Cd, Fe, Mn, Pb, **Zn**	field	1.2	[200]
terrestrial	*Acanthostomum pavidum*, *Archaeodiplostomum acetabulata*, *Protocaecum coronarium*, *Pseudocrocodilicola georgiana*, *P. americana*, *Timoniella loosi*	*Alligator mississippiensis*	l	**As**, **Cd**, **Cu**, **Fe**, Pb, **Se**, **Zn**	field	1–1,154	[30]
	Monogenea						
limnetic	*Ancyrocephalus mogurndae*	*Siniperca chuatsi*	m, l, k, gi	**Pb**	field	na	[34]
	Pentastomida						
terrestrial	*Sabekia mississippiensis*	*Alligator mississippiensis*	l	**As**, Cd, **Cu**, Fe, Pb, **Se**, **Zn**	field	3–399	[30]

*Abbreviations*: *BCF* bioconcentration factors, *bo* bones, *br* brain, *fe* feathers, *gi* gills, *go* gonads, *hr* hard roe, *i* intestine, *k* kidney, *l* liver, *m* muscle, *na* data not available, *sb* swimbladder, *sc* spinal cord, *sk* skin, *te* testes

The individual accumulation potential of various parasite taxa has been investigated in laboratory and field studies. Sures [12] summarized and listed 15 different parasite species which exhibit a high metal accumulation potential. However, the number of studies has increased rapidly in the last decade, and to date more than 50 metazoan parasite species, belonging primarily to the four major endohelminth taxa (Acanthocephala, Cestoda, Digenea and Nematoda) have been considered and suggested as sentinels for metal pollution (see Table 1). Amongst those, cestodes with about 30 different species from different hosts and habitats (limnetic, marine, terrestrial) represent the largest group, followed by nematodes, acanthocephalans and digeneans. Acanthocephalans and cestodes show the highest accumulation capacity so far, being able to accumulate different elements, especially non-essential or toxic ones, at very high levels ([12]; see also Table 1). For example, the concentrations of cadmium and lead have been shown to be up to 2,700 times higher in the acanthocephalan parasite *Pomphorhynchus laevis* than in its hosts’ muscle tissues [23, 24]. Similarly, high levels of these elements were also reported from cestodes, where their concentrations were up to 1,175 times higher compared to host tissues ([25]; see Table 1). Recent studies also demonstrated that cestodes are able to accumulate organic pollutants such as polychlorinated biphenyls (PCB) to a higher degree than their hosts [26]. Elevated levels of different elements were also reported for nematodes, which however mainly accumulate essential elements rather than toxic ones [27, 28]. Accordingly, organisms which take up their nutrients *via* their tegument, such as acanthocephalans and cestodes, appear to be more appropriate sentinels for toxic elements than other parasite taxa which have a gastro-intestinal tract. Laboratory studies on the accumulation of lead suggest that acanthocephalans take up the metal in the form of bile-metal complexes [21]. When exposed to metals, organometallic complexes are formed in the liver of many vertebrate species which then pass down the bile duct into the small intestine where they can either be reabsorbed by the intestinal wall and run through the hepatic-intestinal cycle or they can be excreted with the faeces (see [21] and references therein). If organisms are infected with acanthocephalans the parasites interrupt the hepatic-intestinal cycling of metals, as they were shown to rely on the uptake of bile acids from their host’s intestine [21, 29]. In principle, all substances entering acanthocephalans and cestodes have to pass through their tegument. Accordingly, if substances can be detected in cestodes and acanthocephalans they are biologically available in the sense that they are able to cross biological membranes. Additionally, the parasite’s localization in the host as well as its developmental stage might play an important role in the accumulation process, as the availability of metals differ within the host, and larval parasites exhibit differences in physiology and metabolism in comparison to their adult stages [27, 30].

Studies on the accumulation potential of digeneans are limited and only few species have been investigated to date. However, some species showed a high accumulation capacity [30–32] and an elevated resistance to toxic elements [33], which suggests their possible use as potential sentinels for metal pollution. Interestingly, pentastomids from reptiles also indicate a high accumulation of some essential and non-essential elements [30]. However, published data on this group as well as on Monogenea [34] is still very limited (see Table 1).

Because acanthocephalans, cestodes, nematodes and digeneans are mainly endoparasites without direct contact to the ambient environment, they have access to pollutants through their hosts. As suggested by Sures & Siddall [21], the uptake of metals in a fish-parasite system from freshwater habitats occurs mainly over gills, circulatory system and entero-hepatic circulation of the host. In this way the metals become available for the parasites located in the intestine and other microhabitats within the host. In marine ecosystems, the dietary uptake as well as the uptake from water needed for osmoregulation seem to represent the main sources for metals [19]. Similarly, in terrestrial ecosystems the dietary uptake route of metals is more important than the direct accumulation from ambient environment (e.g. air). Thus, acanthocephalans, cestodes as well as trematodes of terrestrial mammals were also found to accumulate metals in high concentrations in a similar manner as various aquatic parasites [31, 35–37].

Acanthocephalans, cestodes and some nematodes fulfill most of the criteria required for sentinels as suggested by Sures [12]. Most species studied exhibit a high accumulation potential and high resistance to metal pollution (Table 1). Furthermore, most of the species are large in body size, widespread and very abundant in their host and can be easily sampled and identified. Most importantly, pollutant levels in parasites usually correspond to those in the environment. In contrast, other parasite taxa (e.g. monogeneans or different protozoans) do not fulfill some of the main criteria for accumulation indicators. Parasitic protozoans as well as many digeneans and monogeneans are small in size and therefore cannot provide sufficient material for chemical analyses. This might explain the limited (Monogenea, Digenea) information regarding their accumulation potential. However, among the latter group there are also species with larger body sizes and high abundance. Given that high metal accumulation rates were occasionally shown in digeneans (e.g. [30–32]), larger species should be studied more intensively in the future. Due to the direct contact with the ambient environment monogeneans can probably rapidly access and accumulate pollutants and may provide a useful tool if they are large enough.

The use of parasites as additional accumulation indicators requires good arguments in order to compete with the established free-living sentinels, which are much easier to work with. One such argument can be the remarkable accumulation capacity of parasites, as discussed above. Thus, with their help even very low environmental concentrations can be detected and quantified in relatively unpolluted habitats such as the Antarctic (e.g. [38]). Furthermore, sensitive monitoring tools will also be necessary to detect elements with very low natural abundance, such as the technology-critical elements (TCE), which are used in increasing amounts for new technologies. These elements are emitted into the environment through anthropogenic activities, although their environmental behaviour remains largely unclear [39]. Acanthocephalans, for example, are able to accumulate such elements (e.g. Pt, Pd, Rh) at levels above the detection limits of conventional analytic techniques [40]. Furthermore, acanthocephalans and cestodes can be promising organisms for studies addressing the availability of (nano-) particles. If accumulation of elements that were initially in a particulate form occurs in acanthocephalans and/or cestodes, it is necessary that they had to cross several biological membranes [40, 41]. When using filter-feeding organisms such as mussels to study the uptake of particulate elements, it remains unclear if these elements are only adsorbed at the gill filaments or present in the gut content, or if they are really taken up in a biological sense [42]. Parasites could help to close this gap and give a better understanding of the biological availability of pollutants in ecosystems.

## Parasites as pollutant sinks

The enormous accumulation of pollutants in certain parasites can affect the pollutant metabolism of their hosts, as was shown as early as 1996 and 1999 [21, 43, 44]. Using experimental infections and a laboratory exposure experiment with lead, Sures & Siddall [21] reported for the first time that chub infected with the acanthocephalan *Pomphorhynchus laevis* exhibited lower lead concentrations than uninfected conspecifics. This result was confirmed subsequently using the lead isotope ^210^Pb [45]. Likewise, Gabrashanska & Nedeva [43] as well as Turcekova & Hanzelova [44] reported lower metal concentrations in wild fish infected with cestodes compared with uninfected animals. Lower metal levels in acanthocephalan-infected fish were attributed to disturbance of the entero-hepatic cycling of lead within the fish host by the parasite [21]. Successively, a number of studies from different host-parasite systems was published which also showed reduced metal concentrations in tissues of infected hosts from aquatic as well as terrestrial habitats (Table 2). However, contrasting results, where the presence of parasites can increase pollutant burdens in infected hosts, have been described for some host-parasite systems (Table 2). Even if the collection of studies in Table 2 is not complete, it is evident that many cestodes and all investigated acanthocephalans are able to reduce metal levels in different tissues of their hosts. Reasons why the concentrations of the same element were differently affected by *Ligula intestinalis* remain unclear, but may be attributed to the fact that different fish hosts and different elements were studied [46]. It also becomes obvious that there is a strong need for more studies considering possible effects of nematodes and digeneans, as these groups are still understudied in this respect.

**Table 2:** Selected studies describing the effects of parasites on element levels in infected hosts compared to uninfected conspecifics

Habitat	Parasite taxa	Host	Element levels in infected *vs* uninfected hosts	Element	Study type	Reference
	Acanthocephala					
limnetic	*Pomphorhynchus laevis*	*Squalius cephalus*	decrease	Pb	experimental	[11]
		*Squalius cephalus*	decrease	Pb	experimental	[45]
		*Squalius cephalus*	decrease	Cd, Cu, Pb	field	[157]
limnetic	*Acanthocephalus anguillae*	*Squalius cephalus*	decrease	Cd, Cu, Pb	field	[157]
limnetic	*Polymorphus minutus*	*Gammarus roeseli*	decrease	Cd	experimental	[87]
limnetic	*Acanthocephalus lucii*	*Perca fluviatilis*	decrease	Cr, Mn	field	[162]
limnetic	*Acanthogyrus* sp.	*Oreochromis niloticus*	decrease	Pb	field	[164]
	Cestoda					
limnetic	*Bothriocephalus acheilognathi*	*Cyclops strenuus*	decrease	Cd	experimental	[115]
limnetic	*Bathybothrium rectangulum*	*Barbus barbus*	decrease	Cr, Ni, Pb	field	[167]
limnetic	*Ligula intestinalis*	*Alburnus alburnus*	decrease	Cu, Zn	field	[43]
		*Rastrineobola argentea*	decrease	Cu	field	[46]
		*Rastrineobola argentea*	increase	Cd, Cr, Zn	field	[46]
limnetic	*Proteocephalus macrocephalus*	*Anguilla anguilla*	decrease	Cr, Ni	field	[184]
limnetic	*Proteocephalus percae*	*Perca fluviatilis*	decrease	As, Cd	field	[44]
limnetic	*Proteocephalus percae*	*Perca fluviatilis*	decrease	Pb	field	[162]
limnetic	*Proteocephalus percae*	*Perca fluviatilis*	decrease	Cr, Mn	field	[162]
marine	*Clestobothrium crassiceps*	*Merluccius merluccius*	decrease	As, Cd, Hg, Pb	field	[170]
terrestrial	*Mesocestoides* spp.	*Vulpes vulpes*	decrease	Pb	field	[178]
terrestrial	*Mesocestoides* spp.	*Vulpes vulpes*	increase	Cu, Mn	field	[178]
terrestrial	*Moniezia expansa*	*Ovis aries*	decrease	Pb	experimental	[179]
terrestrial	*Hymenolepis diminuta*	*Rattus norvegicus*	decrease	Cd, Zn	experimental	[202]
terrestrial	Unidentified cestodes	*Sterna paradisaea*	decrease	Bi	field	[203]
	Nematoda					
terrestrial	*Toxascaris leonina*	*Vulpes vulpes*	decrease	Pb	field	[178]
limnetic	*Raphidascaris acus*	*Oncorhynchus mykiss*	decrease	Se	experimental	[204]
estuarine	*Eustrongylides* sp.	*Fundulus heteroclitus*	decrease	Hg	field	[205]
	Digenea					
limnetic	Different digeneans	*Littorina littorea*	decrease	Cu, Fe, Ni, Pb	field	[206]
	Isopoda					
estuarine	*Probopyrus pandalicola*	*Palaemonetes pugio*	decrease	Hg	field	[205]

**Fig. 1:**
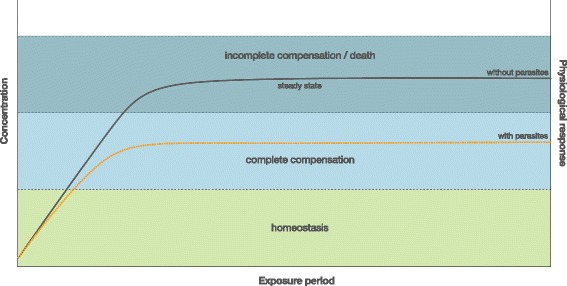
Accumulation kinetics showing the concentration of a toxic substance in tissues of infected and uninfected hosts. At the steady state concentration, the uptake and elimination rates of the substance are balanced. The accumulation of toxic substances is associated with the physiological response of the exposed organism, i.e. at lower tissue concentrations physiological responses allow for a complete compensation of adverse effects. Thus, if the level of the steady state concentration is reduced due to parasitism, less severe toxic effects can be expected for the host compared to uninfected conspecifics

A possible reduction of pollutant concentrations in infected hosts has important implications. Pollutant accumulation in organisms can be assumed to result from a balance of different uptake and loss mechanisms depending on the infection status. The uptake by parasites has to be considered as an efflux from the fish host, similar to elimination [47] and can therefore directly reduce the steady state concentration of the host (Fig. 1). If animals are sampled from the field for environmental monitoring programs, pollutant levels in infected hosts can thus be lower compared to uninfected specimens. If data from infected and uninfected animals are not separated, there will be a high degree of variation. If, on the other hand, mainly infected organisms are analysed, pollutant concentrations in a given habitat are probably underestimated due to parasite-reduced tissue concentrations. This highlights the need to consider the complete host-parasite system, rather than just the host (or the parasite) alone, for such monitoring and pollution assessments. An interesting question for future research would be the ecosystem relevance of pollutant accumulation in parasites. The question arises if and how parasites alter pollutant dynamics within food webs and how this affects the health of the interacting organisms.

## Parasite effects on biomarkers and host physiology

**Fig. 2:**
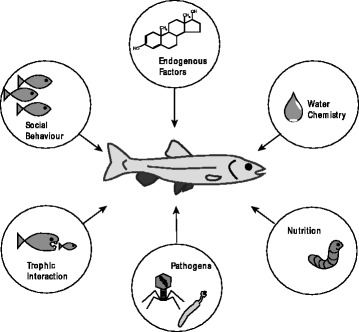
Physiology, biochemistry and behaviour of organisms is affected by various internal and external parameters (drawing by Dr. Nadine Ruchter). Citation: Sures B, et al. [207] Biological effects of PGE on aquatic organisms. In: Zerein F, Wiseman CLS, editors. Platinum metals in the environment. Heidelberg: Springer Berlin Heidelberg; pp. 383–399. With permission of Springer

Physiological responses of organisms to pollutants are a consequence of the uptake and accumulation of toxic substances (Fig. 1). The variety of responses ranges from an increased level of stress and protective molecules to a complete breakdown of physiological homeostasis and death of the exposed organism. A common approach in ecotoxicology is to use responses on a biochemical or molecular level as early warning signs to indicate the presence of contaminants and to unravel possible adverse effects on organisms [48, 49]. These responses, commonly defined as biomarkers, are analysed in environmental monitoring programs using different free living animals, such as molluscs (e.g. [50]), crustaceans (e.g. [51]) and fish (e.g. [52]), amongst others. The most commonly used biomarkers refer to measures of oxidative stress, hormone regulation, energy budgets, as well as genes and proteins involved in pollutant metabolism and excretion. Accordingly, these biomarkers are usually not a specific response to pollutants but might rather be induced by a variety of other stressors, including parasites [17, 53]. Additionally, contaminant specific markers are used for monitoring, which indicate the presence and effects of specific pollutants, such as metallothioneins as markers for metals [54], or the induction of cytochrome P4501A that is used as a specific biomarker for exposure in fish to aryl hydrocarbon receptor (AhR) agonists such as polycyclic aromatic hydrocarbons (PAHs), pesticides and polychlorinated biphenyls (PCBs) [52]. Under environmental conditions, however, organisms are not only exposed to pollutants but are also confronted with a variety of other endogenous and exogenous factors (Fig. 2). Accordingly, the extent to which biomarkers are able to provide unambiguous and ecologically relevant indication of exposure to or effects of toxicants remains highly controversial [49]. Forbes et al. [49] therefore stressed that biomarkers may most successfully be used for hypothesis generation in controlled experiments and that more efforts are needed to develop models of appropriate complexity that can describe real-world systems at multiple scales in order to apply the biomarker concept under field conditions.

There has been an increasing awareness in recent years that parasites strongly interact with pollutant-induced biomarker responses of their hosts by influencing their physiology in a multitude of different ways. There are two main approaches, the first being experimental exposure to contaminants and parasites in the laboratory, and the second being measurement of biomarker responses in fish infected with differing numbers of parasites from both polluted and reference conditions [53]. As it is not possible to review all the studies investigating combined effects of pollutants and parasites on physiological responses of their hosts (but see [53, 55–57]), examples of recent studies highlight the main categories of results usually described. The studies vary depending on the parasite and host species chosen as well as on the pollutant investigated [53, 56], and the outcome of a parasite-pollution interaction would either lead to reduced (e.g. [58]) or increased levels of biomarkers (e.g. [59]). These interactions have two main important implications: they affect the reliability of biomarkers as a diagnostic tool to determine the presence and effects of pollutants [53]; and they are the physiological basis for possible adverse effects on the hosts [56]. Both aspects are briefly summarized below. In general, the prediction can be made that larval parasites in intermediate hosts requiring trophic interactions for transmission should be more virulent [60] and thus lead to increased pathology in combination with contaminant stressors.

Modulation of biomarker responses in organisms being simultaneously infected with parasites and exposed to environmental pollutants is a phenomenon which is currently not well understood and which deserves further investigation. In certain cases, a biomarker response may increase or decrease, making interpretation difficult. For example, with biomarkers of oxidative stress it is advisable to use several enzymes and substrates involved in oxidative stress metabolism as well as pathological endpoints to better understand the stress response and physiological effect on the host [61]. Markers of energy metabolism such as total lipid and glycogen content were also shown to be differentially modulated by parasitism. Although no effect on glycogen levels due to Cd exposure were detected in uninfected gammarids, infection with microsporidians led to higher glycogen concentrations [62, 63]. Levels of heat-shock proteins (HSP) as indication of a general stress response in organisms are usually increased due to pollutants but may be significantly reduced when exposed gammarids are infected with acanthocephalans [58, 64]. In contrast, microsporidian infections may lead to a pronounced heat shock response [63]. Also, pollutant-specific markers such as metallothioneins (MT) were found to be sensitive to modulation by parasites. Digenean parasites in Cd-exposed cockles lead to a decrease in MT concentrations compared to uninfected individuals [65, 66].

Moreover, it appears that effects commonly considered to result from environmental pollution can partly be attributed to parasites. Anthropogenic endocrine active compounds present in surface waters are an example of major environmental concern due to their potential health effects on the reproductive system in aquatic vertebrates [67, 68]. In addition to chemicals, infection with parasites can also affect the development of gonads in different groups of animals, such as crustaceans and fish (e.g. [69–73]). The most intensively studied model organism with respect to its effects on host’s gonad development and the reproductive status is the larval cestode *Ligula intestinalis*. Infection of fish with *L. intestinalis* has long been known to inhibit reproduction in this second intermediate host [74, 75]. It was demonstrated that inhibition of gametogenesis in infected roach (*Rutilus rutilus*) was accompanied by a pronounced disruption of the hypothalamus-pituitary-gonad (HPG) axis, which is the prime endocrine system regulating reproduction [76–79]. With respect to possible biomarkers it was shown that plasma concentrations of sex-steroids as well as the expression of gonadotropins in the pituitary were lower in infected fish than in uninfected [76–78]. This example shows that endocrine disruption of reproductive biology in fish is not only caused by natural and synthetic substances but also by naturally occurring parasite infections. However, the degree to which parasitism contributes to endocrine disrupting phenomena of wildlife remains unknown and therefore deserves further investigations.

This collection of examples shows that parasites might modulate the expression of various biomarkers. If such biomarkers are analysed as part of monitoring programs to identify environmental pollution, false-negative as well as false-positive results can be obtained. In addition, laboratory assays using biomarkers are typically performed with uninfected hosts, so extrapolation of effects to natural conditions where most animals are parasitized is problematic and underestimates the actual severity of polluted conditions [53]. Accordingly, manipulation of biomarker response by parasites easily leads to misinterpretation of pollution scenarios, if parasitism is not considered. We therefore have to get a more detailed picture of the interaction between parasites and pollutants, which means that research in this field of EP has to be intensified. Furthermore, in most study systems, there has not been strong corroboration between field and laboratory results [57]. Additionally, an increased use of modern “omic”-techniques would probably help in getting a more detailed and mechanistic understanding of the possible interactions.

The variety of interactions between parasites and pollutants may also directly affect the health of the host in different ways [17, 18, 53, 55, 56]. For example, several environmental pollutants may suppress the immune response of organisms, thereby leading to higher parasite infection intensities (e.g. [53, 55, 56, 80, 81]; see also chapter below). On the other hand, parasites themselves may also change the physiological or biochemical response of the host to a pollutant in different directions as both stressors might interact in a synergistically, antagonistically or additive way [53, 56, 82]. Accordingly, the health of organisms simultaneously confronted with parasites and pollutants can be more or less seriously threatened as compared to confrontation with either stressor alone [53]. However, some contrary cases have also been reported in which parasites appear to be beneficial to their host. In the following, a selected number of representative studies will be presented that are suitable to represent the variety of results that can be expected.

From a theoretical point of view, one would expect less severe effects if the steady state concentration of a pollutant in an exposed organism is reduced, e.g. due to parasites as shown by the examples listed in Table 2. Additionally, a reduced toxicity might also result if other physiological pathways are triggered by parasites that lead to changes of the host’s pollutant metabolism. In fact, a couple of studies have shown positive effects of parasites on selected life parameters. Recently, Sánchez et al. [83] demonstrated that parasites can increase host resistance to arsenic. In acute toxicity tests using *Artemia parthenogenetica* infected with the larval cestode species *Flamingolepis liguloides* and *Confluaria podicipina* infection consistently reduced mortality across a range of arsenic concentrations and at different temperatures. Infected *A. parthenogenetica* had higher levels of antioxidant enzymes as well as a higher number of carotenoid-rich lipid droplets, both of which help to reduce oxidative stress. Heinonen et al. [84] showed that freshwater clams, *Pisidium amnicum*, infected with digenean trematode larvae were less sensitive to pentachlorophenol (PCP) and survived longer than uninfected conspecifics. However, it should be mentioned that even if infected clams were able to survive longer under exposure conditions than uninfected conspecifics, they cannot reproduce due to the castrating effects of the digeneans. From a fitness’ point of view, the prolonged survival of infected molluscs is therefore of no advantage for the host. In general, it remains an interesting question, why parasites lower their hosts’ toxic burdens. From an evolutionary perspective, this seemingly altruistic behaviour towards their host could have developed as a strategy to keep the host alive under stressful conditions, as the demise of the host also interrupts the parasite life-cycle.

Exposure experiments have been conducted in gammarids infected with different acanthocephalan larvae with equivocal results. Until recently, it was commonly accepted that gammarids infected with acanthocephalan larvae, mainly *Pomphorhynchus laevis* and *Polymorphus minutus*, suffer more during exposure studies with metals than uninfected individuals (e.g. [58, 85, 86]). However, Gismondi et al. [87] presented results which suggest that infections with *P. minutus* could be advantageous for *Gammarus roeseli* during Cd exposure. When studying sex-specific lethality of Cd, LC_50_ values revealed that infected *G. roeseli* males showed lower mortality under cadmium stress than uninfected ones. The opposite result, however, was found for female gammarids. A slightly higher mortality (although not significant) in cadmium-exposed, uninfected *Gammarus fossarum* compared to *P. minutus*-infected individuals was also found by Chen et al. [63]. The mechanisms by which an acanthocephalan infection in gammarids can potentially be beneficial remain largely unclear. Independently of gender, unexposed infected *G. roeseli* had lower protein and lipid contents but higher levels of glycogen [59]. Increased glycogen levels in acanthocephalan-infected gammarids seems to be a common phenomenon [63] and might result from an increased uptake of nutrients due to extended energy requirements [88]. Under polluted conditions the need for detoxification of pollutants can cause increased metabolic activity. Gismondi et al. [62] described an increase of several host antitoxic defence capacities in *P. minutus*-infected *G. roeseli* females following cadmium toxicity although infection increases cadmium toxicity in *G. roeseli* females.

Examples showing additive negative effects of parasites and pollutants are more frequently found than beneficial effects. For example, Gheorgiu et al. [89] demonstrated a strong increase in mortality if guppies (*Poecilia reticulata*) were simultaneously exposed to Zn and infected with the monogenean *Gyrodactylus turnbulli*. Also for other fish species as well as amphibians many examples exist which show that pathogenicity of parasites may be enhanced under polluted conditions, many of which represent a field-based approach [53, 64, 82, 90–93]. In summary, most papers suggest a worsening of pollution-induced adverse effects by parasites. However, it cannot be excluded that parasite-reduced pollutant concentrations in infected hosts might be beneficial when hosts face environmental pollution, as lower pollutant levels are usually associated with less toxic effects. This assumption has to be studied in more detail in future investigations with a clear focus on the question if negative effects of a parasitosis may be outweighed by a potentially positive impact of reduced pollutant levels. When tackling these aspects, further consequences on the population and ecosystem levels should be addressed.

## Contaminant effects on free-living stages of parasites

In addition to a possible use of parasites as accumulation indicators several studies also focus on effect indication with parasites on the level of individual organisms, populations and communities. Effect indication on the individual level might be possible using the direct toxicity of substances on free-living parasitic stages, mainly those of digeneans such as miracidia and cercariae (reviewed in [94]). Within these studies a known number of larval stages were treated with chemicals, mainly metals, and the subsequent longevity, viability and infectivity of the stages were analysed [55, 94]. This research was done with two goals in mind: first, to determine if studies of effects of pollution on free-living stages could help evaluate changes in parasite populations in polluted waters, and second, to develop the use of free-living stages of parasites, in particular, the asexually-produced cercariae emerging from infected molluscan hosts, as sensitive toxicity indicators. However, little work has been aimed at evaluating the effects of pollution on infectivity of free-living stages of parasites [94]. Since that time, there has not been much advancement in this area of research. Most of the activity focused on agricultural systems and the effects of pesticides and eutrophication, with varying results. For example, atrazine has been shown to have negative effects on echinostome cercariae, impeding transmission and decreasing infection levels, but also negative effects on anuran tadpole hosts, increasing susceptibility and infection levels [95]. Various pesticides were shown to cause mortality of echinostome cercariae, yet sublethal exposures did not reduce infectivity [96]. These authors also found that pesticide exposure increased susceptibility of tadpoles to infection to a greater degree than it impeded cercarial transmission, resulting in net increases in infection [96]. In another study, cercarial mortality was increased and survival decreased to varying degrees in two trematodes exposed to different concentrations of atrazine [97]. Cercariae of another echinostome suffered reduced survival following exposure to each of six different insecticides, but not in a dose-dependent manner [98]. This calls into question the potential use of toxicity assays in predicting pesticide toxicity to parasites [98]. In contrast to these studies, echinostome eggs and miracidia were not affected by exposure to any of four pesticides [99]. In a fish study, infectivity of two diplostomatid cercariae was reduced following exposure to cadmium, even at low doses for short time periods [100].

Aside from trematodes, and also infecting anurans, another parasite attracting attention is the chytrid fungus, responsible for numerous amphibian declines and extinctions worldwide [101]. Three different pesticides tested inhibited zoospore production, while two of three inhibited zoosporangia development, and all three caused mortality of both fungal stages [102].

Parasite responses to eutrophication, another byproduct of agricultural activity, were very different from those to pesticides and other contaminants by comparison. Infections of *Ribeiroia ondatrae*, a trematode responsible for frog malformations, increased following eutrophication (see also section on Ecosystem health). Not only did eutrophication lead to an increased density of infected snails, but it resulted in an increase in the per-capita cercarial production by infected snails [103].

With respect to a possible use of free-living stages as sensitive toxicity indicators several studies showed a reduced infectivity and longevity of cercariae. Following the first reports on the sensitivity of cercariae to metal ions published by Evans [104, 105] a possible application of metal ions was tested as a treatment against cercariae of disease-causing *Schistosoma* sp. (e.g. [106–109]). In the meantime, miracidia and cercariae of many other trematode species were tested for their sensitivity to metal pollution in laboratory exposure studies [55, 94]. However, compared with conventional effect indication procedures, such as the automatic mussel monitor [110], cercarial test systems appear to be less applicable as toxicity indicators. Moreover, due to the relatively short life span of cercariae, a monitoring system using these organisms would be too complicated to be routinely used.

## Parasites as indicators of ecosystem health

Undoubtedly, parasites are important and integral elements in aquatic ecosystems in which they drive fundamental ecological processes, e.g. by contributing to a system’s biodiversity, productivity and food web structure or ecosystem engineering (e.g. [111, 112]). A healthy, i.e. functioning and resilient [113], ecosystem is therefore a system rich in parasite species [13, 114]. As with free-living species, parasites respond to ecosystem disturbances and can provide valuable information about a system’s quality, integrity and health in response to pollutants and other stressors. Other than the use of parasites as accumulation indicators, e.g. when assessing the biological availability of pollutants, environmental impacts are usually assessed *via* changes in an organism’s behaviour or numbers, which acts as an effect indicator [94]. However, just like free-living taxa, different parasite taxonomic groups react differently to environmental impacts (see meta-analyses by Lafferty [10]; Blanar et al. [14]; Vidal-Martínez et al. [15]). It is therefore important that suitable parasite taxa are chosen as bioindicators in accordance with the research question, and that different parasite taxonomic groups are not randomly pooled when analysing environmental stressors [13, 15]. However, grouping parasites into different functional groups has been shown to provide useful results and insights, e.g. when comparing monoxenous (single host life-cycle) and heteroxenous (multi-host life-cycle) parasites, endo- and ectoparasites, allogenic and autogenic parasites, planktonic *vs* benthic life-cycles, or species sharing common life-cycle pathways [13, 14].

Ectoparasites and free-living parasite dispersal stages (e.g. cercariae, coracidia) show clear parallels to free-living animals, since they are in direct contact with their environment and toxic substances can directly affect them, reducing their vitality or increasing mortality (see above [94, 115]), and thus leading to changes in composition and diversity of parasite communities. For example, the occurrence of monogenean ectoparasites on fish as well as the hatchability and survival of their larvae was found to be negatively affected by high metal concentration in water [116–118]. Exposure to Zn also reduced reproduction and survival of *Gyrodactylus turnbulli* on guppies [119]. Curiously, maximum intensities of *G. turnbulli* on guppies were observed at low to moderate concentrations, but declined at higher levels of Zn [89]. These results highlight the differential impact of contaminants on parasites and their hosts, affecting the dynamics of the host-parasite relationship. Endoparasites on the other hand live within their host and environmental effects usually manifest first at the level of the host and subsequently can be detected at the parasite level. Altogether, environmental pollution was shown to have generally stronger, mostly negative, effects on directly exposed parasites (i.e. free-living stages and ectoparasites) when compared with endoparasites [14].

In contrast to monoxenous parasites, heteroxenous parasites require one or more hosts for their transmission and their distribution is dependent on the presence of all hosts in their life-cycle [13, 112, 120]. Higher pollutant exposure concentration and duration might perturb the life functions of the host (intermediate, paratenic or final), leading to lower reproduction rates and/or higher mortality, which should reduce host population size. Under certain conditions, hosts of other parasites may proliferate, leading to increases in the transmission of their parasites, as can be observed with eutrophication [13]. As mentioned above, pollutants also can directly affect free-living stages of parasites, reducing their populations [94]. Given that the different groups of parasites have life-cycles that use various components of the food web for transmission, changes in composition and diversity of heteroxenous parasite communities can provide information about the environmental impact on the food web that led to the disruption or enhancement of the transmission of the various parasites in that community [13, 112]. Because of this direct linkage of heteroxenous parasites to the free-living host communities at different trophic levels, these organisms are considered sensitive bioindicators for aquatic ecosystem health [13, 112].

However, as stated above, meta-analytical approaches have shown that monoxeneous taxa tend to show a higher susceptibility to a larger variety of environmental stressors [14]. Does this mean that monoxeneous endoparasite taxa are the most sensitive and reliable bioindicators? This would certainly be an overgeneralization, given the specific and sometimes contradicting responses even within functional groups. The following recent studies therefore provide some examples of promising parasitic bioindicators that do not fit to this general pattern.

Digenean trematodes offer promising effect indicators due to their complex life-cycles with a first intermediate molluscan host and a wide variety of definitive hosts (e.g. birds, mammals, amphibians), as well as second intermediate hosts, such as fish, molluscs, insects or crustaceans that are all necessary to complete and maintain the life-cycle. Field investigations have shown that the prevalence of digeneans in their intermediate and definitive hosts are inversely related to the degree of pollution and disturbance of aquatic ecosystems ([121] and references therein), and trematode diversity indices might work as well as established insect diversity indices to assess ecosystem health [122, 123]. Furthermore, due to their complex multi-host life-cycles, trematodes are reliable indicators of free-living species diversity [124] and can reveal the trophic interactions within an ecosystem.

Also the composition and diversity of total parasite communities of a particular host (e.g. fish) were found to reflect the ecological condition of habitats where the host occurs. For example, Nachev & Sures [125] and Chapman et al. [126] reported higher parasite diversity at less polluted sampling sites, whereas the composition of the parasite fauna and the abundance of some parasites showed a clear relationship with the pollution gradient. Furthermore, various studies demonstrate, for example, that toxic pollution reduces the diversity of heteroxenous parasites, whereas parasites with direct life-cycles (monoxenous) are less affected (e.g. [127]), despite the results of the meta-analyses described above. The proliferation of certain monoxenous parasites likely is due to an impaired host immune response in polluted conditions [13, 112]. Given that monoxenous parasites show a different susceptibility to pollution than heteroxenous ones, the ratio between species richness of heteroxenous and monoxenous (S_H_/S_M_) parasites can be used as a measure of pollution impact, showing distinctively higher S_H_/S_M_ ratios on/in fish in unpolluted marine habitats in comparison to fish sampled from polluted habitats ([127] and references therein). While species composition and richness did not change along a pollution gradient in a northern Canadian river, the relative abundance of a monoxenous monogenean ectoparasite increased and then decreased, while that of a heteroxenous larval trematode decreased then increased as the river coursed through a heavily-impacted mining area [128].

In using parasites as indicators of ecosystem stress and environmental degradation, it is important to disentangle effects caused by contaminants from those caused by other abiotic or biotic factors. For example, in a study examining effects of municipal effluents in a large North American river, fish parasite communities were affected primarily by water mass, year and season [129]. This study highlights the importance of good sample design and replication. This point is supported by other studies which showed large-scale hydrological effects on parasite communities to be more pronounced than those of contaminants [130–132]. Indeed, Marcogliese et al. ([129] and references therein) suggested that parasite communities as indicators may not be sensitive enough to detect effects of low to moderate pollution or that effects may be overshadowed by those of natural environmental variation. This reinforces the point above that circumstances will dictate the choice of parasite indicator, be it a particular taxon, guild or functional group [13]. This is nicely illustrated by another study in the same river and on the same fish species as in [129], replicated over 5 years, that showed the species richness and prevalence of myxozoan parasites in the same fish increased downstream of a large municipal effluent [130]. The study further demonstrated that the effects were due to eutrophication leading to the proliferation of oligochaete alternate hosts and not contaminant toxicity.

Another important development of the last decade where there has been a wealth of research has been the examination of the effects of pesticides on anuran parasites in North America and their interaction, largely spurred forward by the controversy over the herbicide atrazine, which is banned in Europe [133, 134], as well as the marked increase in reports of malformations in frogs [135]. These studies have examined the effects of pesticides experimentally in mesocosms as well as in the field, focusing on either selected parasite species, trematodes as a whole, or entire communities [103, 136–143]. Two general patterns have emerged from these studies. First, there is renewed interest in eutrophication and disease [144–146]. Secondly, parasite communities appear to reflect the landscape structure of their hosts’ habitat. A variety of studies examining the effects of pesticides on frog parasite communities have independently found that abundance of trematodes or parasite species richness are positively correlated with the surrounding forest area, and negatively correlated with the amount of urban or agricultural area [137–143]. Landscape also was associated with changes in community structure in fishes in streams and rivers [128, 147]. Another significant step forward from these initiatives is the application of an experimental mesocosm approach to examine the effects of pesticides on host-parasite interactions within simulated communities, allowing the disentanglement of direct toxic effects and indirect ecological effects along with the mechanisms involved [103, 141]. Furthermore, manipulation of parasite life-cycles within the laboratory also has permitted the determination of the mechanisms involved in direct and indirect effects of pesticides on host-parasite interactions [96].

Apart from measuring and indicating anthropogenic ecosystem disturbances, such as pollution and degradation, parasites can help to monitor and assess the redevelopment and restoration of ecological habitats that were converted into more ‘productive’ or useful systems in the past. These changes and alterations of landscapes and aquatic systems have resulted in extensive degradation of ecosystems and the loss of biodiversity on a global scale [148]. The last decades have seen the implementation of rehabilitation and restoration schemes to reverse such degradation and recreate the original/natural (or near-natural) state of ecosystems. Measuring the success and impacts on a restored ecosystem and its biodiversity is crucial to assess such actions and benthic macroinvertebrates and fishes are commonly used as bioindicators [148]. Additionally, heteroxenous parasites with complex life-cycles, such as trematodes, have proven to be suitable assessment tools for the restoration of waterways and wetlands and have been shown to reflect the level of restoration success (e.g. [13, 123, 149]). However, despite their promising application, only few studies have applied snail-trematode systems to assess the effect of aquatic restoration projects to date and more studies should address this potentially very interesting field of research in the future to obtain a more complete picture of the free-living and parasite diversity and community structure in these systems.

While the number of studies using or including parasites as indicators to assess ecosystem health is slowly but steadily increasing, we certainly need more studies to unravel and better understand the different and sometimes opposing effects of environmental impacts on different parasite taxa. Also, as Vidal-Matínez et al. [15] point out, it is important to establish the threshold at which certain parasite groups respond to environmental impacts. Selected parasite groups show the high potential as indicators of ecological health, when chosen according to the research question and when studies are carefully designed; e.g. trematode communities in aquatic habitat restoration assessments [123, 149], or the use of heteroxenous and monoxenous parasite ratios as indicators of pollution impact [127]. As Blanar et al. [14] conclude, the final choice of indicator taxa should be based on local ecology, parasite biology, and the specific research question that is to be addressed.

Lastly, resource managers and scientists should not rely on a single pollution indicator. The use of parasites as indicators should be combined with other indicators to obtain the most comprehensive understanding of the pollution problem in question [13, 150, 151]. For example, Vidal-Martínez et al. [152] examined contaminant levels and parasites within the same individual shrimp hosts. Another study utilized chemical measurements of the sediments, plus physiological biomarkers and parasites from the same individual fish [153]. Most recently, a study in a large river measured metals in surface waters, along with stable isotopes, condition, histological examination, transcriptomic and biochemical analyses and selected parasites in the same fish [154, 155]. Marcogliese et al. [156] summarizes a multidisciplinary research program, including parasitology, aimed at examining the effects of a major municipal effluent on the same large river ecosystem.

## Conclusions

The research directions reviewed here show that parasites can be considered as organisms whose responsiveness might be advantageous to the understanding of environmental problems. Apart from highlighting promising research directions and identifying future research needs, the current paper should make non-parasitologists aware of parasites by exemplifying the sensitivity of these organisms to environmental changes as well as by focusing on the physiological effects parasites may have on their hosts. The selected examples and ideas on the other hand may enable parasitologists to consider and treat parasites not only as creatures that threaten the health of their hosts but also as responsive organisms with applied bioindication value in an environmental sense.
